# Sustainable Valorization of Agro-Industrial Waste and Polymer Residues for High-Plasticity Clay Stabilization: An Agro-Rubber Hybrid Approach

**DOI:** 10.3390/polym18151907

**Published:** 2026-08-03

**Authors:** Fahad Alshawmar, Bisma Khalid, Sana Ullah, Waqas Hassan, Mudassir Mehmood, Sofia Sarwar

**Affiliations:** 1 Department of Civil Engineering, College of Engineering, Qassim University, Buraydah 51452, Saudi Arabia; 2 Department of Transportation Engineering and Management, University of Engineering and Technology, Lahore 54890, Pakistan; bisma.khalid@unsw.edu.au; 3 School of Engineering and Technology, UNSW Canberra, Canberra, ACT 2600, Australia; 4 Department of Civil, Environmental, and Infrastructure Engineering, College of Engineering and Computing, George Mason University, Fairfax, VA 22030, USA; sanaullahuet978@gmail.com; 5 Geotill Engineering Inc., Fishers, IN 46038, USA; waqashasan65@gmail.com; 6 Sinosteel Maanshan General Institute of Mining Research Co., Ltd., Maanshan 243000, China; 7 NUST Institute of Civil Engineering, National University of Sciences and Technology (NUST), Islamabad 44000, Pakistan; sofiasarwar2020@gmail.com

**Keywords:** eggshell powder, rubber tire powder, expansive soil, stabilization, mechanical properties, additives, sustainability, polymer

## Abstract

Expansive soils, characterized by pronounced volumetric instability under moisture fluctuations, pose a persistent challenge to the safety, serviceability, and long-term stability of civil infrastructure. In response to these challenges, increasing attention has been directed toward the use of sustainable waste-derived additives as environmentally responsible alternatives for improving problematic soils. This study investigates the mechanical behavior of expansive soil stabilized with waste eggshell powder (ESP) and recycled tire powder (RTP), an elastic polymeric waste material rich in rubber and carbon black derived from discarded tires. A comprehensive experimental program was conducted to evaluate the combined effects of ESP/RTP on soil performance. For this purpose, Atterberg’s limits, compaction, unconfined compressive strength (UCS), Swell potential, and California bearing ratio (CBR) tests were performed on untreated soil and soil modified with up to 25% ESP and 9% RTP. The experimental findings revealed that the incorporation of ESP and RTP significantly reduced the Atterberg limits, optimum moisture content (OMC), and swelling potential of the soil, with the optimum blend of 20% ESP and 6% RTP yielding the most pronounced overall improvement. Such changes signify a considerable reduction in soil plasticity and expansive susceptibility, thereby enhancing the volumetric stability of the treated soil. At the same time, maximum dry density (MDD), UCS, and CBR values increased appreciably, demonstrating improved compaction behavior, greater mechanical resistance, and superior load-bearing performance. Moreover, one-way ANOVA confirmed that the enhancements in UCS and CBR achieved at the optimum mix proportions were statistically significant at the 95% confidence level. Overall, the findings demonstrate that ESP and RTP can serve as a sustainable, cost-effective, and environmentally friendly stabilization solution for improving the engineering performance of expansive soils.

## 1. Introduction

Expansive soils, which undergo significant swelling and shrinking, are among the most challenging soil types encountered in civil engineering. Their pronounced volumetric instability can significantly impair the performance and service life of foundations, pavements, embankments, and other infrastructure systems, often resulting in structural damage, repeated maintenance, and considerable economic loss [1,2]. The complex and uncertain behavior of these soils poses serious challenges to site investigation, design, and construction. While conventional stabilization methods have long been employed to control their detrimental effects, such techniques are often associated with high financial costs and environmental concerns. In recent years, therefore, growing emphasis has been placed on sustainable stabilization approaches, particularly the utilization of waste materials and polymer-based additives, which not only improve the engineering behavior of problematic soils but also contribute to resource recovery and environmental sustainability in construction practice [3,4].

Among these sustainable additives, rubber tire powder (RTP), obtained from discarded waste tires, has gained notable attention due to its dual environmental and engineering significance. The large volume of end-of-life tires generated worldwide poses a serious waste-management challenge because of their non-biodegradable nature, long-term persistence, and potential environmental and public-health risks when improperly disposed of. Converting such waste into fine rubber powder offers a sustainable reuse pathway that not only mitigates disposal problems but also produces a polymeric additive capable of improving soil flexibility, toughness, and resistance to cracking under loading conditions [5,6]. The polymeric structure of RTP gives it remarkable elasticity, durability, and resilience under load. When incorporated into soil, RTP particles form an interlocking matrix with soil grains, improving load-bearing capacity and reducing susceptibility to deformation and cracking [7,8]. Consistent with these favorable characteristics, a growing body of research has demonstrated the effectiveness of RTP in soil stabilization. For instance, Akbarimehr, Eslami [9] investigated how mixing waste tire rubber with Tehran clay affects soil properties. Adding about 10% rubber improved strength and made the soil less brittle, while higher amounts (up to 50%) reduced density but created lighter, more flexible mixtures that can be useful as filler materials in construction projects. Similarly, Yang, Lu [10] examined how rubber content and particle size affect the dynamic behavior of expansive soil-rubber mixtures. Their results showed that 10% rubber content with 0.25 mm particles provided the best dynamic performance, while higher rubber content altered stress responses, and different soil-rubber interactions were identified to explain the behavior under freezing conditions. Additionally, Akbarimehr et al. [9]] reported that the same proportion reduced compaction effort, making soil preparation more efficient. Baldovino, Diaz [11] further confirmed that cement-stabilized soils benefited from the addition of rubber powder, showing improvements in compressive strength and overall load-bearing properties. Likewise, studies combining rubber powder with other materials, such as seashell ash or cement and lime, also reported notable enhancements in soil stiffness and durability [12,13,14]. Collectively, these studies highlight the considerable potential of RTP as both a sustainable waste-reuse material and a functional additive for enhancing the engineering performance of problematic soils, including expansive soils.

In parallel, eggshell powder (ESP), a byproduct of the egg-processing industry, has emerged as a sustainable soil stabilizer. Primarily composed of calcium carbonate along with trace minerals and organic matter, ESP exhibits natural cementitious properties, which improve soil structure and promote particle aggregation [15,16]. When incorporated into expansive soils, calcium-bearing phases from ESP may interact with reactive silica and aluminosilicate constituents present in the soil under alkaline conditions, contributing to secondary cementitious products such as calcium silicate hydrates (C-S-H) and calcium aluminate hydrates (C-A-H). Additionally, ESP can improve soil performance through filler effects, particle aggregation, and changes to soil structure. Several studies have demonstrated that even small percentages of ESP can significantly enhance the geotechnical properties of soils, making it an effective and environmentally friendly stabilization method [17,18].

Under favorable conditions, eggshell powder may facilitate physicochemical interactions with reactive soil minerals, leading to secondary cementitious reactions due to its high calcium content. In addition to filler action and cation exchange, stabilization behavior is also influenced by improved particle bonding within the soil matrix. [19]. By reinforcing the soil structure and increasing the soil’s bearing ability, these hydrated chemicals function as binding agents [20]. Anoop, Beegom [21] utilized eggshells up to 2% as a replacement for lime in soil stabilization. Their results showed a remarkable increase in soil strength with the use of eggshell powder. Alzaidy, Mohammed NJ [22] incorporated eggshell powder of up to 8% and plastic waste of up to 1% to stabilize the soil. They revealed an increase in bearing capacity and a decrease in the soil’s swelling pressure. Anburuvel, Sathiparan [23] analyzed the increase in the strength properties of soil by utilizing eggshell powder and rice husk ash in expansive soil. A similar study conducted by Zada, Haleem [20] demonstrated an increase in the strength properties of expansive soil by adding eggshell powder up to 5%, along with a reduction in the swelling potential and soil plasticity. Paul et al. [24] studied expansive soil stabilized with eggshell powder up to 20% and quarry dust up to 30%, indicating an increase in the CBR and shear strength of the expansive soil. Recently, interest in sustainable soil stabilization using waste-derived, environmentally friendly materials as alternatives to conventional chemical stabilizers has grown significantly [25,26]. Modern sustainable binders, including industrial byproducts, geopolymers, biomass ash, and polymer-based additives, have shown considerable potential to improve the engineering behavior of problematic soils while supporting resource recovery and environmental sustainability [27,28,29,30]. Recent studies have further demonstrated the effectiveness of hybrid waste stabilization systems in improving strength, reducing plasticity and swelling behavior, and enhancing the long-term performance of stabilized soils through environmentally friendly construction [31,32,33,34].

However, although the individual contributions of ESP and RTP to soil stabilization have been widely reported, their combined influence on the engineering behavior of expansive soil has received comparatively limited research attention. In this context, this study investigates the combined effect of waste materials ESP and RTP for improving the strength and volumetric behavior of expansive soil within a sustainable geotechnical engineering framework. The significance of combining ESP and RTP lies in their complementary stabilization mechanisms. Owing to its high calcium carbonate content, ESP contributes to enhanced strength and reduced plasticity [35], while RTP improves flexibility and reduces cracking [36]. Their combined application is therefore expected to provide a more balanced and effective stabilization response than the use of either additive alone. Accordingly, this study aims to clarify the synergistic effects of ESP and RTP on the engineering performance of expansive soil and to evaluate their potential as sustainable alternative stabilizers for geotechnical applications.

## 2. Materials and Methods

### 2.1. Materials

#### 2.1.1. Soil

**Figure 1:**
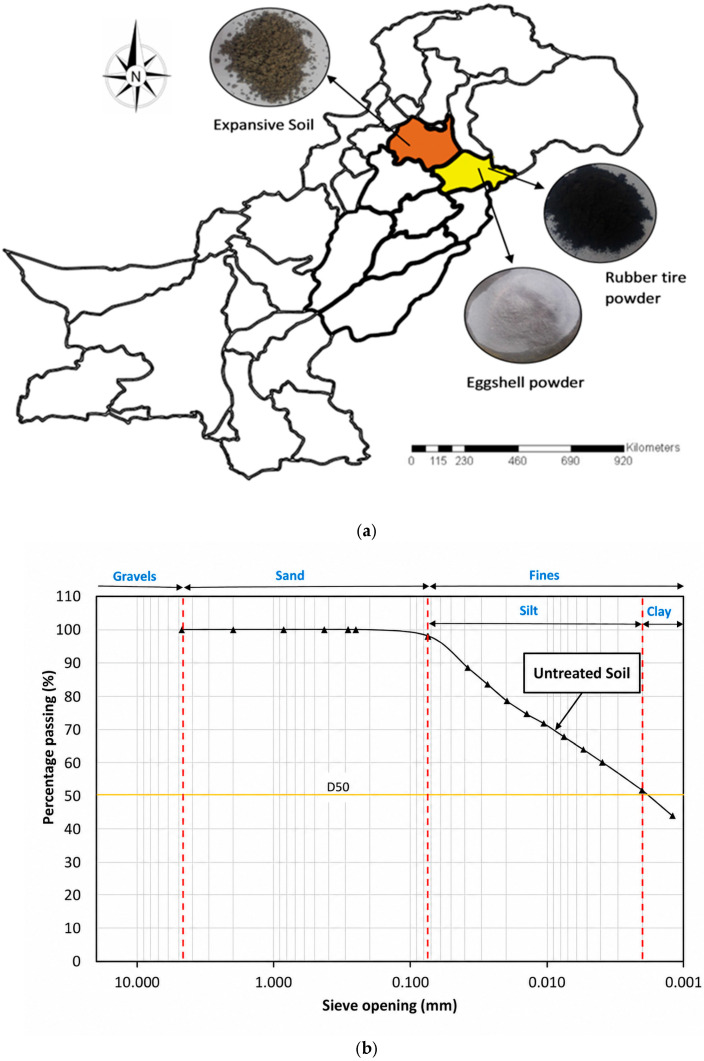
(**a**) Location of study area. (**b**) Particle size distribution of untreated soil. Dashed vertical lines mark the boundaries between gravel, sand, silt, and clay fractions. The horizontal line indicates D50, the particle diameter at 50% passing.

Expansive soil was collected from Nandi Pur, Lahore, Pakistan, as shown in Figure 1a. Table 1 and Table 2 present the physical and chemical parameters of the expansive soil. Particle-size distribution was determined by sieve and hydrometer analyses using ASTM compliant test sieves, a mechanical sieve shaker, a soil hydrometer, a sedimentation cylinder, and a dispersion mixer (Humboldt Mfg. Co., Elgin, IL, USA), in accordance with ASTM D6913 and ASTM D7928, as shown in Figure 1b. The liquid limit (LL) of 62% and plastic limit (PL) of 29% were determined using a liquid-limit device, an ASTM grooving tool, and a plastic-limit test set (Humboldt Mfg. Co., Elgin, IL, USA), in accordance with ASTM D4318. The unconfined compressive strength (162 kPa) was determined using an unconfined compression loading frame with a constant rate-of-strain attachment and a dial gauge (Humboldt Mfg. Co., Elgin, IL, USA), in accordance with ASTM D2166. The maximum dry density (1.32 g/cm^3^) and optimum moisture content (23%) were determined using a standard Proctor mold, a 5.5 lb (2.5 kg) rammer, and a sample extruder (Humboldt Mfg. Co., Elgin, IL, USA), in accordance with ASTM D698. Swelling potential (63%) was measured using a fixed-ring consolidometer, porous stones, and a dial gauge (Humboldt Mfg. Co., Elgin, IL, USA), in accordance with ASTM D4546. The unsoaked and soaked California bearing ratio values (2.3% and 1.2%, respectively) were determined using a CBR load frame, penetration piston, and surcharge weights (Humboldt Mfg. Co., Elgin, IL, USA), in accordance with ASTM D1883. The soil was then classified in accordance with the Association of State Highway and Transportation Officials (AASHTO) standard as A-7-6 and with the Unified Soil Classification System (USCS) as fat clay (CH).

#### 2.1.2. Eggshell Powder

The process of preparing ESP involved multiple steps to ensure cleanliness, proper drying, and fine grinding. First, waste eggshells were collected from the cafeterias of the University of Engineering and Technology, Lahore, Pakistan. To remove any remaining yolk, membrane, or other residues, the eggshells were thoroughly rinsed under running water. Once cleaned, they were evenly spread on aluminum foil and placed in an oven at 93 °C for 30 min [46]. Temperatures of 93 °C were used primarily for sterilization and moisture removal. This study did not consider higher calcination temperatures because excessive heating may alter the chemical composition and reactivity of calcium carbonate in ESP, a topic beyond its scope. After oven drying, the eggshells were further air-dried at room temperature to remove any remaining moisture.

**Figure 2:**
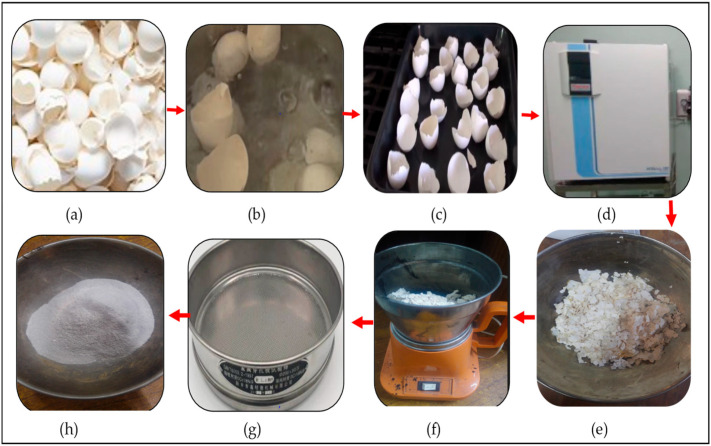
Physical procedure for producing eggshell powder: (**a**) raw eggshells; (**b**) washing; (**c**) air-drying; (**d**) oven-drying; (**e**) crushed shells; (**f**) crushing/grinding; (**g**) sieving; (**h**) final eggshell powder.

When the eggshells were completely dried, they were transferred to a clean grinder for pulverization into a fine powder. Grinding was done intermittently to prevent the grinder from overheating, which could alter the properties of the powder. After grinding, the eggshell powder was passed through a 100-mesh sieve to ensure uniform particle size and to remove any large particles [19]. Finally, the finely ground eggshell powder was stored in a sealed glass jar to prevent contamination and unwanted chemical reactions with external substances. The entire physical process of producing eggshell powder is visually represented in Figure 2, while its chemical composition is detailed in Table 2.

#### 2.1.3. Rubber Tire Powder

**Figure 3:**
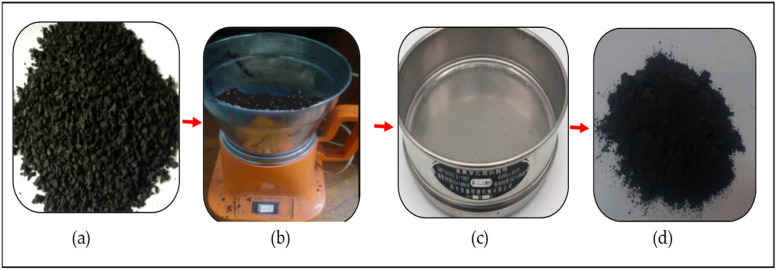
Physical procedure for making rubber tire powder: (**a**) shredded rubber tire pieces; (**b**) grinding; (**c**) sieving; (**d**) final rubber tire powder (RTP).

RTP involved several steps to ensure proper size reduction and removal of large particles. First, shredded pieces of discarded rubber tires were collected from an automotive repair shop in Lahore, Pakistan. These rubber pieces were then fed into a grinder to break them down into smaller, more manageable fragments. After grinding, the rubber tire particles were passed through a sieve with a mesh size of 40. This sieving process helped separate the finer powder from any remaining larger particles, ensuring uniformity in the final product. The sieved material, now in powder form, was collected as RTP. This entire procedure for producing rubber tire powder is visually depicted in Figure 3, while Table 3 provides detailed information on the physical and chemical properties of the processed rubber tire powder.

### 2.2. Test Sample Preparations

**Figure 4:**
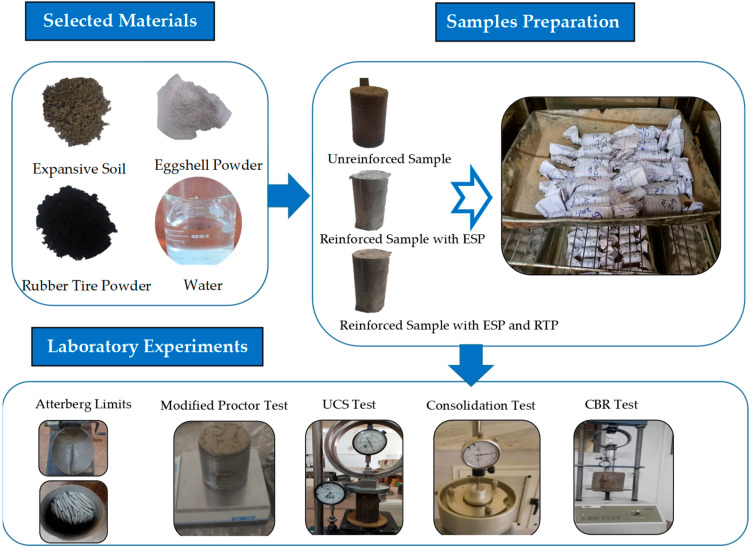
Experimental workflow showing material selection, specimen preparation, curing, and laboratory testing.

The test specimens were prepared by homogeneously mixing the soil with specified proportions of ESP and RTP. According to the additive composition, the prepared specimens were categorized into three experimental groups for subsequent laboratory evaluation. All laboratory tests were conducted in triplicate, and the reported values represent the average of three measurements. The first group consisted of unreinforced soil samples, which were pure soil with no additives, serving as the control group. The second group included soil samples reinforced with ESP, where the ESP was added at varying percentages of 10%, 15%, 20%, and 25%. The third group consisted of soil samples reinforced with a combination of ESP and RTP. In this group, the optimal amount of ESP was combined with RTP, with the percentage of both materials ranging from 3% to 9%. Figure 4 illustrates the methodology flowchart that shows how the samples were categorized and the laboratory tests that followed.

The maximum dosages of ESP (25%) and RTP (9%) were selected based on two considerations. First, the selected dosage ranges were informed by comparable studies in which ESP and RTP contents of up to 20% were investigated [9,24]. Second, dosage increments in the present study were extended beyond the point of peak performance to identify the onset of a downward trend. At 25% ESP and 9% RTP, the compaction and strength parameters, including MDD, UCS, and CBR, had already declined relative to the 20% ESP and 6% RTP optimum (Section 3.2, Section 3.3 and Section 3.5), indicating that the optimum had been exceeded within the investigated dosage range and that further increases were unlikely to yield additional improvement.

### 2.3. Laboratory Experiments

#### 2.3.1. Modified Proctor Test

The modified Proctor test was conducted according to ASTM D1557 standards. Five kilograms of dry, expansive soil were used for the experiment. The soil was passed through a 19-mm sieve. Prior to adding water, the dry soil was carefully mixed with the required proportions of ESP and RTP to achieve a homogeneous blend. Subsequently, water was added to the soil and thoroughly mixed to achieve a uniform mixture when reinforced with ESP and RTP. A similar procedure was also adopted by [32,33,52,53,54]. The soil mixture was then molded into five layers. Each layer was compacted using a 4.89 kg rammer with 25 blows and a fall height of 450 mm. To ensure uniformity during the Modified Proctor test compaction, each soil layer was compacted with a standard number of blows from a hammer of specified weight, dropped from a consistent height. Before adding a new layer, the surface of the previous layer was scratched to promote uniform distribution of compaction effects. This process was repeated for each layer to maintain consistent compaction throughout the sample. For rammers, this involved verifying mass, drop height, and dimensional accuracy. Gauges were calibrated by comparing readings against known reference standards and adjusting as necessary. The compacted soil was then extracted from the mold to obtain the optimum value of moisture content and maximum dry density.

#### 2.3.2. Unconfined Compressive Strength Test

The UCS test was conducted following ASTM D2166 standard procedures. Samples were prepared by adding different ratios of ESP and RTP, along with the optimum value of water content attained from the Modified Proctor tests. The samples removed from the mold had dimensions of 100 mm in height and 50 mm in diameter. Both untreated and treated samples were then cured for 0, 14, and 28 days, with the samples wrapped to maintain moisture levels. The UCS of different reinforced and unreinforced soil samples was then observed with a strain rate of 1 mm/min [55,56].

#### 2.3.3. One-Dimensional Consolidation Test

A one-dimensional swell test was performed in line with ASTM standard D4546 specifications to measure swelling potential. The swelling potential was determined using an oedometer apparatus. The procedure involved placing a compacted specimen, with a diameter of 5 cm and a height of 2 cm, within a ring. The empty weight of the ring was calculated using a balance. Filter sheets were then placed between the stone and specimen, at the bottom and top of the assembly. Next, the assembly was submerged in water, and the gauge reading was set to zero. A pressure of 48 kPa was applied, and the dial gauge readings were recorded at uniform intervals with respect to vertical displacement. During the test, the specimen was allowed to swell freely, and its volume changed accordingly. The swell pressure, indicating the pressure at which the specimen swells, was measured. The dial gauge values were taken at regular intervals until the expansion variations reached zero. The percentage swell was determined using the given formula.

% swell = Δh/h

where Δh and h represent the change in height and the original height of the soil sample, respectively.

#### 2.3.4. California Bearing Ratio Test

The soaked and unsoaked CBR tests were conducted following ASTM D1883 standards. Sample preparation involved considering the optimum moisture content and maximum dry density achieved through a modified Proctor test [57]. A plunger with a diameter of 5 cm was employed to apply a penetration rate of approximately 12.5 cm/min. For the soaking CBR value determination, the samples underwent immersion in a water tank. Both soaked and unsoaked CBR values were obtained using a surcharge mass of 2.5 kg after various curing periods of 28 days.

### 2.4. Statistical Analysis (SA)

Statistical analyses were performed using the three replicate measurements obtained for each treatment condition. The effect of ESP was evaluated using untreated soil and soil containing 5%, 10%, 15%, 20%, and 25% ESP. The effect of RTP was assessed at 0%, 3%, 6%, and 9% RTP while maintaining the ESP content at 20%. Each soil parameter and curing period was analyzed separately. The normality of the model residuals was examined using the Shapiro–Wilk test, while equality of variance among the treatment groups was assessed using Levene’s test. When both assumptions were satisfied at *p* > 0.05, the groups were compared using one-way ANOVA. A significant overall ANOVA result was followed by Tukey’s honestly significant difference test to identify the treatment groups that differed. When residual normality was not satisfied, the Kruskal–Wallis test was applied, followed by Dunn’s pairwise test with Holm-adjusted *p*-values when the overall test was significant. Welch’s ANOVA and the Games–Howell test were reserved for cases in which the assumption of equal variance was not satisfied. Statistical significance was evaluated at α = 0.05. Effect sizes were calculated as eta squared (η^2^) for one-way ANOVA and epsilon squared (ε^2^) for Kruskal–Wallis analyses, as given in Equations (1) and (2), respectively.

(1) $$\eta^{2}=\frac{{SS}_{between}}{{SS}_{total}}$$

(2) $$\epsilon^{2}=\frac{H-k+1}{n-k}$$

where *η*^2^ and *ε*^2^ are eta-squared and epsilon-squared, the effect size measures for one-way ANOVA and the Kruskal–Wallis test, respectively; *SS* = sum of squares; *H* = the Kruskal–Wallis test statistic; *k* = the number of groups being compared; and *n* = the total number of observations across all groups. Statistical analyses were performed using Microsoft Excel, Version 2506 (Microsoft 365, Microsoft Corporation, Redmond, WA, USA), and OriginPro 2026 (OriginLab Corporation, Northampton, MA, USA).

## 3. Results and Discussion

### 3.1. Effect of ESP and RTP on Soil Atterberg Limits

**Figure 5:**
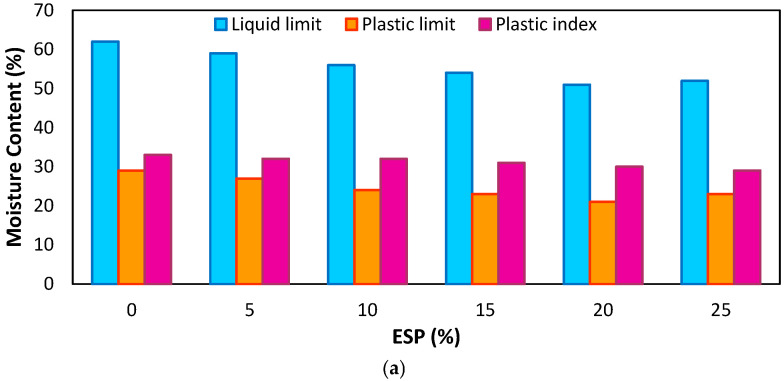

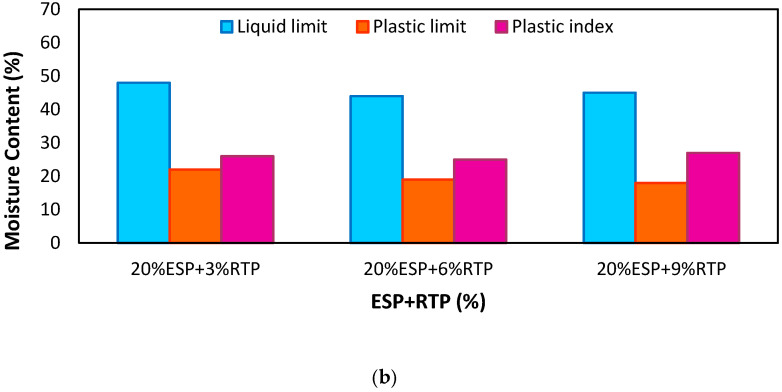
Atterberg limits (liquid and plastic limits) values for (**a**) both unreinforced soil and soil reinforced with ESP and (**b**) soil reinforced with ESP and RTP.

Figure 5a illustrates the Atterberg limits (liquid and plastic limits) values for both untreated and treated soil with ESP. The ESP content ranges from 0% to 25%, with increments of 5% added to the unreinforced soil sample. The maximum decrease in liquid and plastic limits is observed when using 20% ESP in the soil. The figure illustrates a decrease in the liquid limit from 62% to 51% and the plastic limit from 29% to 21% by using 20% ESP. After that, further addition of ESP helps in the reduction in both liquid and plastic limit values. Therefore, 20% was taken as the optimum dosage for incorporating ESP. However, plasticity continued to decrease slightly at 25% ESP, and the reduction beyond 20% ESP was relatively small. Therefore, 20% ESP was selected as the optimum dosage for subsequent evaluation with RTP inclusion.

In Figure 5b, the enhancement in the Atterberg limits of expansive soil is depicted by incorporating the optimal dosage of ESP (20%) with different RTP ranges of 3%, 6%, and 9%. It is observed that with the utilization of 20% ESP and 6% RTP, there is a maximum decrease in the liquid limit from 62% to 44% and the plastic index value from 33% to 25%. However, with further increases in RTP content, the Atterberg limits continue to increase. For example, Barazesh et al. [44] analyzed a decrease in the liquid limit of expansive soil from 69% to 58% by utilizing 24% ESP. On the other hand, Surjandari and Dananjaya [45] demonstrated that by utilizing ESP up to 20%, there was a decrease in the liquid limit from 110% to 77%, and the plastic index decreased from 67% to 29%. The study conducted on Tehran clay soil analyzed the enhancement in the Atterberg limits of soil reinforced with rubber tire powder [46]. Conversely, the study on Australian expansive soil examined the decrease in Atterberg limits when reinforcing the soil with rubber tire powder [19].

### 3.2. Effect of ESP and RTP on Compaction Properties

**Figure 6:**
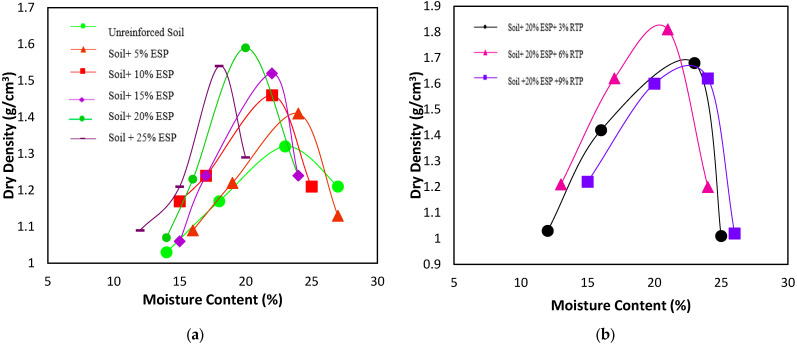

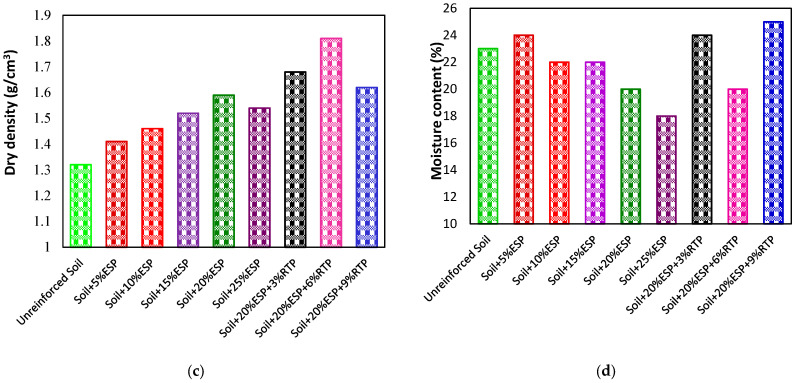
Relationship between moisture content value and dry density of (**a**) unreinforced soil and soil reinforced with ESP, (**b**) soil reinforced with optimum ESP with RTP, (**c**) MDD of unreinforced and reinforced soil, (**d**) OMC of unreinforced and reinforced soil.

Figure 6a illustrates the relationship between moisture content value and dry density of modified soil and natural soil reinforced with ESP up to 25% with 5% increments. Figure 6b illustrates the relationship between water content and dry density of soil reinforced with the optimum values of ESP and RTP up to 9% with 3% increments. The figures demonstrate that dry density increases and moisture content decreases with an increase in ESP up to 20% and RTP up to 6%.

Figure 6c shows that the MDD of unreinforced and reinforced soil ranges from 1.32 g/cm^3^ to 1.81 g/cm^3^. The MDD of unreinforced soil is 1.32 g/cm^3^. The maximum value of MDD while incorporating 20% ESP (the optimum value) into the soil is 1.59 g/cm^3^. The maximum dry density achieved by incorporating 6% RTP with the optimum value of ESP is 1.81 g/cm^3^.

Figure 6d displays the OMC of unreinforced and reinforced soil, ranging from 18% to 25%. The OMC of unreinforced soil is 23%. The minimum value of OMC while incorporating 25% ESP into the soil is 18%. The minimum OMC achieved by incorporating 6% RTP with the optimum value of ESP is 20%. Notably, at 25% ESP, MDD decreased from 1.59 g/cm^3^ (20% ESP) to 1.54 g/cm^3^, and at 9% RTP, MDD decreased from 1.81 g/cm^3^ (6% RTP) to 1.62 g/cm^3^. This reversal at the highest tested dosages indicates that the optimum had already been exceeded within the investigated range and suggests that further dosage increases were unlikely to provide additional improvement.

When ESP or RTP particles are combined with soil, they efficiently occupy the empty spaces inside the soil structure. This results in an improvement in the compact density of the soil mixture [58]. In addition, ESP and RTP particles are impermeable, indicating that they do not soak up water like particles found in natural soil. As a result, the existence of these substances decreases the ability of the soil mixture to hold water [59,60]. Consequently, the necessary moisture content for compaction reduces, resulting in a reduced moisture value in the compacted soil mixture. The alterations in density and moisture content enhance the engineering characteristics of the soil blend, rendering it appropriate for various construction purposes, including road construction, embankment stabilization, and slope reinforcement.

### 3.3. Effect of ESP and RTP on Unconfined Compressive Strength

**Figure 7:**
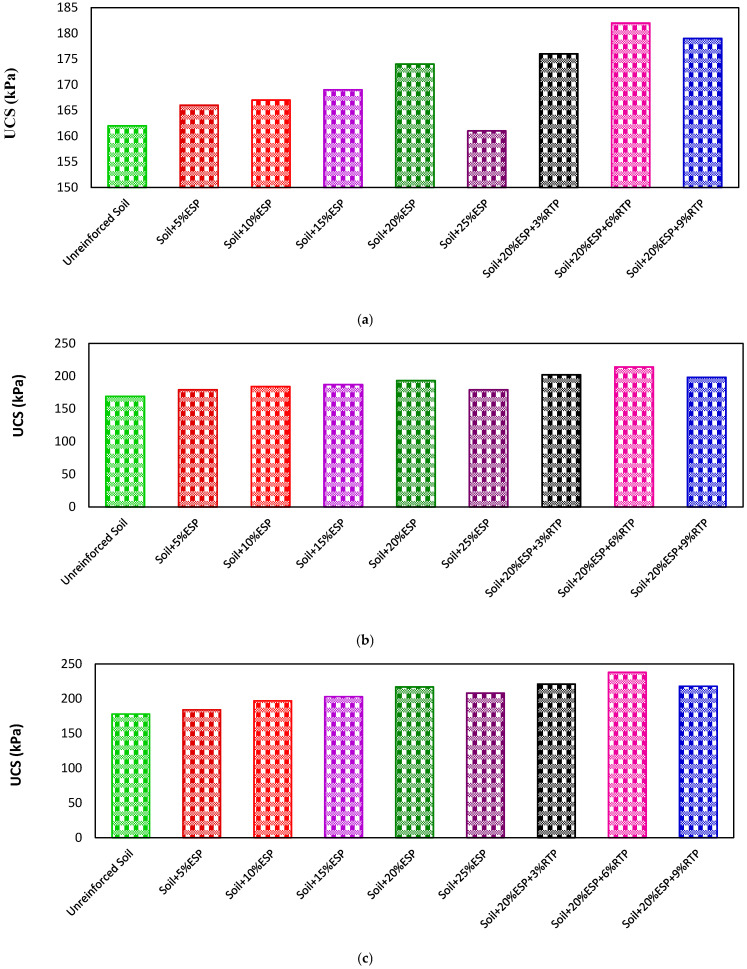
Unconfined compressive strength results of unreinforced and reinforced soil with ESP and RTP at (**a**) 0 days, (**b**) 14 days, and (**c**) 28 days.

Figure 7a illustrates the UCS at 0 days. For unreinforced soil, the UCS is 162 kPa. Incorporating ESP up to 25% in 5% increments shows the maximum improvement at 20% ESP, where the UCS increases from 162 kPa to 174 kPa. Adding RTP at 3% increments, the optimal enhancement occurs at 6% RTP combined with 20% ESP, raising the UCS from 162 kPa to 182 kPa. Figure 7b presents the UCS at 14 days. The unreinforced soil has a UCS of 169 kPa. With 20% ESP, the UCS improves from 169 kPa to 193 kPa. The addition of 6% RTP with 20% ESP further increases the UCS from 169 kPa to 214 kPa. Figure 7c shows the UCS at 28 days. The unreinforced soil exhibits a UCS of 178 kPa. At 20% ESP, the UCS rises from 178 kPa to 217 kPa. Combining 6% RTP with 20% ESP elevates the UCS from 178 kPa to 238 kPa. A combination of 6% RTP and 20% ESP increases the UCS from 178 kPa to 238 kPa. The significant increase in UCS measured at 28 days was primarily due to progressive cementitious and pozzolanic reactions with ESP during curing, while RTP also contributed by enhancing interlocking and stress distribution within the dense soil matrix. These observations indicate that both ESP and RTP considerably improve the UCS of the soil, with the most noticeable improvements recorded at 20% ESP and 6% RTP curing, while RTP also contributed through enhanced interlocking and stress distribution within the dense soil matrix. These observations indicate that both ESP and RTP considerably improve the UCS of the soil, with the most noticeable improvements recorded at 20% ESP and 6% RTP.

Surjandari and Dananjaya [61] observed a similar phenomenon by utilizing ESP up to 15% in fine-grained soil. Another study showed an enhancement in the UCS value of expansive soil by utilizing ESP up to 4% [20]. Harikaran, Kulanthaivel [62] analyzed the combined effect of lime and ESP in increasing the UCS value of soil, with the maximum improvement seen by utilizing ESP up to 12% and lime up to 9% in expansive soil. Similar phenomena have been studied by other researchers on expansive soil by adding ESP [63,64,65]. Naseem, Mumtaz [66] investigated the combined effect of RTP and cement on the UCS of dredged soil. The results showed the maximum improvement in UCS by utilizing RTP up to 15% with an optimum cement value of 2%. A study on alluvial soil showed an improvement in UCS value by adding RTP up to 20% [67]. Another study on black cotton soil analyzed the improvement in UCS value by adding RTP up to 4% and stone dust up to 20% [68]. ESP and RTP particles enhance the UCS of the soil by improving its mechanical properties. ESP, rich in calcium carbonate, acts as a binding agent, filling voids and reducing soil porosity, thus increasing density and strength [69]. RTP particles add flexibility and toughness to the soil, improving ductility and reducing brittleness [70]. This combination creates a stronger, more durable composite soil, providing a sustainable solution for soil stabilization. However, the absolute UCS gain may appear moderate relative to the additive dosage; this should be interpreted in the context of the soil type and stabilization objectives. The test soil is a high-plasticity fat clay (CH), which is inherently resistant to large strength gains due to its dense microstructure and high clay mineral activity. More importantly, the primary engineering concern for expansive soils is volumetric instability rather than UCS alone. In this regard, at the optimum dosage, the swelling potential was reduced by over 80%, and the soaked CBR improved by 300%, both of which are critical performance indicators for expansive subgrade soils. The combined improvement in strength, volumetric stability, and load-bearing capacity, achieved using waste-derived materials at negligible raw material cost, confirms the practical preference for this approach over conventional chemical stabilizers for expansive soil applications.

**Figure 8:**
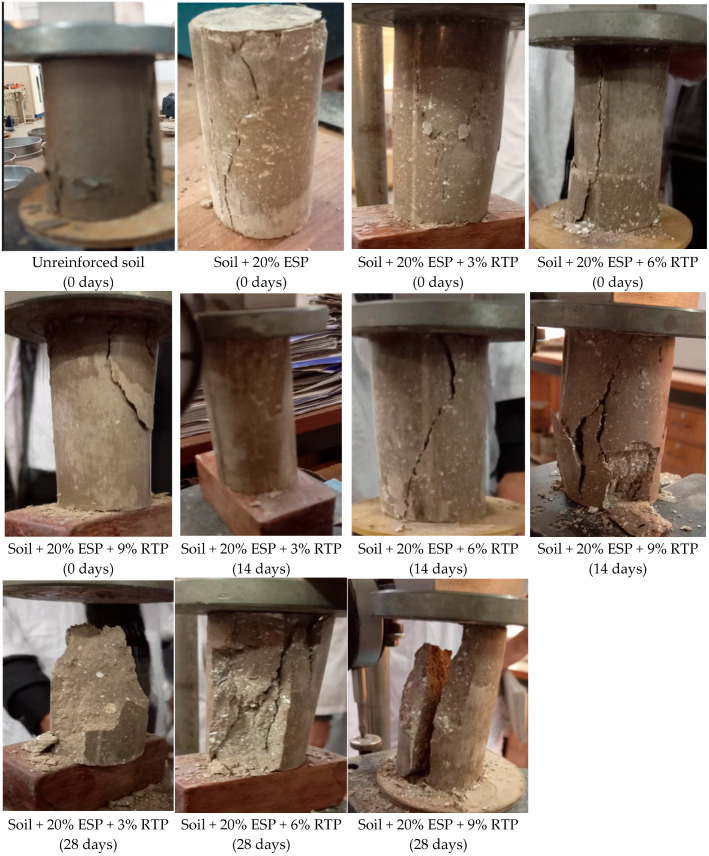
UCS test results of cracked samples of unreinforced and reinforced soil.

Figure 8 shows the UCS test results of cracked samples of unreinforced and reinforced soil. Various types of fracture patterns observed in soil UCS tests can provide useful information on the soil’s behavior and features under stress. Vertical cracks indicate a quick rupture with little deformation, indicating strong compressive strength but moderate flexibility [71]. Horizontal or lateral fractures, on the other hand, may suggest a more ductile failure, in which the soil deforms significantly prior to failure [72]. Diagonal or shear fractures that occur at an angle typically indicate a combination of compressive and shear stress failure, implying that the soil has intermediate qualities, balancing strength and ductility [73]. Furthermore, a variety of crack forms may occur, indicating complex interactions within the soil structure. The type and orientation of these fractures help engineering and geotechnical professionals evaluate the soil’s strength, cohesiveness, and general stability, which is important for planning foundations and other structures.

### 3.4. Effect of ESP and RTP on Swell Potential

**Figure 9:**
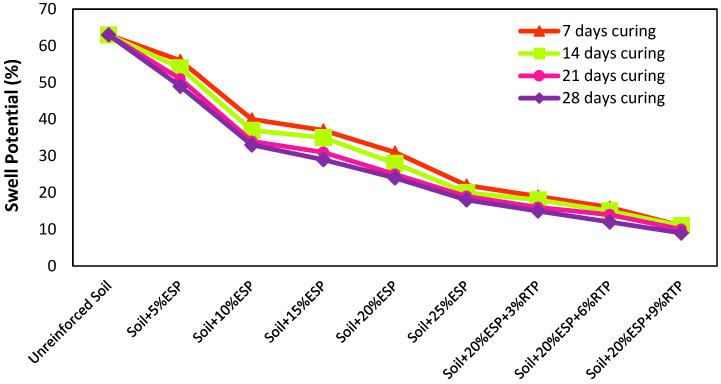
Swell potential of unreinforced and reinforced soil at different curing periods.

Figure 9 shows the effect of ESP and RTP on the swelling behavior of soil after 7, 14, 21, and 28 days. The swelling potential continues to decrease up to 20% ESP + 9% RTP, reaching an 85.7% reduction relative to untreated soil at 28 days, compared with 80.9% at 20% ESP + 6% RTP (Figure 9). However, this additional 4.8% gain in swell reduction at 9% RTP is accompanied by a decline in compaction and strength performance: MDD improvement drops from 27.1% to 18.5%, and 28-day UCS improvement drops from 32.6% to 22.5%, relative to 6% RTP. Because swelling potential at 6% RTP is already reduced to a low, practically manageable level, this trade-off does not favor 9% RTP as the optimum dosage; 20% ESP + 6% RTP is therefore retained as the optimum blend and is used as the basis for all subsequent sections. The high initial swelling potential recorded in the untreated soil suggests the presence of highly active expansive clay minerals and indicates the natural soil’s high volumetric instability under soaking conditions. For instance, Alzaidy [22] observed a similar phenomenon through the combined effect of plastic waste and ESP. The maximum decrease in swelling potential was observed when ESP was utilized up to 8%. Another study on expansive soil utilized ESP up to 5% and also observed a decrease in soil swell potential [20]. Prasad K, Nagendra [59] analyzed the combined effect of ESP with rock dust, noting a decrease in swell potential by utilizing ESP up to 4% and rock dust up to 30%. Naseem, Mumtaz [66] investigated the combined effect of RTP and cement kilns on the swell potential of expansive soil. The maximum reduction in swell potential was observed by utilizing RTP up to 5% and cement kilns up to 10%. Similar research has also analyzed the phenomenon of RTP in reducing the swell potential of expansive soil [74,75].

The significant reduction in swelling potential observed in this study can be explained by three interconnected mechanisms. First, calcium ions released from ESP during hydration replace the highly hydrophilic sodium and potassium ions on clay mineral surfaces via cation exchange. This ion exchange compresses the diffuse double layer around clay particles, directly reducing their water adsorption capacity and tendency to swell [19,76]. Second, the formation of C-S-H and C-A-H products within soil pore spaces fills voids that would otherwise allow water ingress and volumetric expansion. As these cementitious products accumulate during curing, the swelling potential continues to decrease, consistent with the time-dependent reduction observed at 7, 14, 21, and 28 days, as shown in Figure 9. Third, RTP particles physically block inter-aggregate pore channels within the soil matrix, restricting water movement into the clay structure and limiting the degree of saturation that drives swelling. The combined action of these three mechanisms—cation exchange by ESP, pore filling by cementitious products, and physical pore blocking by RTP explains why the ESP–RTP system achieved an 80.9% reduction in swelling at the optimum dosage, a result that significantly exceeds what either additive produces independently. It is acknowledged that direct microstructural validation via SEM, XRD, or FTIR was beyond the scope of this study; future work should include such characterization to further confirm the proposed mechanisms.

### 3.5. Effect of ESP and RTP on California Bearing Ratio

**Figure 10:**
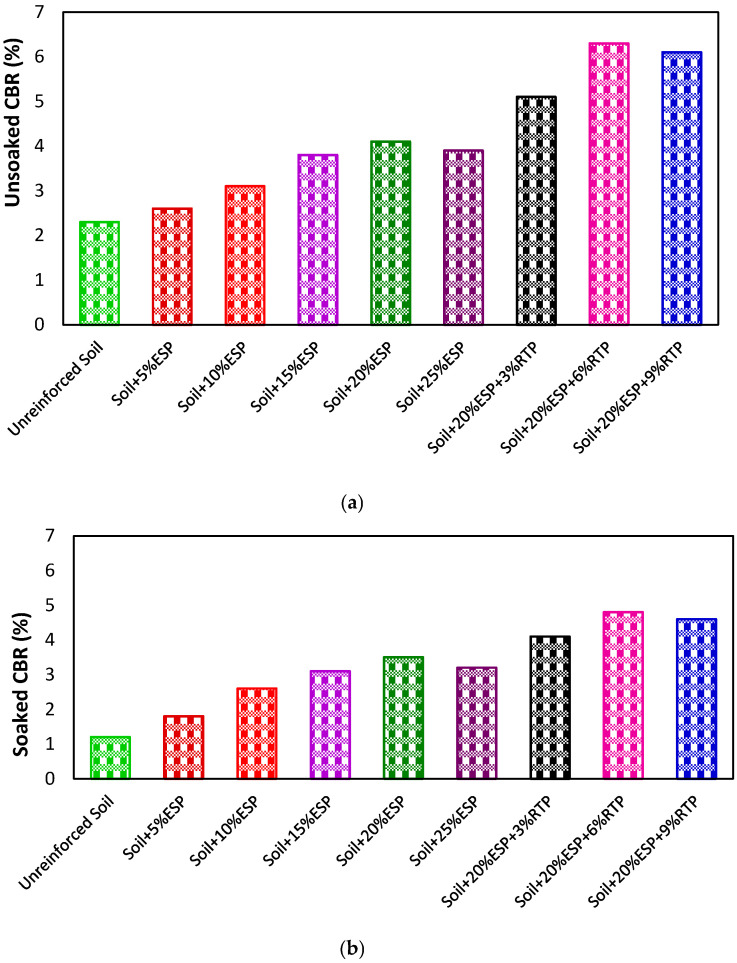
California bearing ratio test for unreinforced and reinforced soil (**a**) unsoaked and (**b**) soaked (28 days).

Figure 10 shows the effect of ESP and RTP on the unsoaked and soaked CBR values. The CBR values for unreinforced soil are 2.3 for the unsoaked test and 1.2 for the soaked test. Figure 10a illustrates the effect of ESP and RTP on the unsoaked CBR value. From the figure, there is an improvement in the CBR of the soil, with an increase in ESP of up to 20% and RTP of up to 6%. The maximum improvement in the unsoaked CBR value, reaching 6.3, is observed with the utilization of 20% ESP and 6% RTP. Figure 10b depicts the effect of ESP and RTP on the soaked CBR value after 28 days. The figure shows an improvement in the bearing ratio of the soil, with an increase in ESP up to 20% and RTP up to 6%. The maximum improvement in the soaked CBR value, reaching 4.8, is observed with the utilization of 20% ESP and 6% RTP.

A similar phenomenon was observed by Carlina, Apriyanti [76], who used bagasse ash up to 13% and ESP up to 3%. There was an increase in the CBR value of clayey soil from 4.93 to 8.61 at 10 blows in an unsoaked condition. Another study noted the same effect, showing that by adding ESP up to 13%, there was an improvement in the CBR value from 6.47 to 9.52 in an unsoaked condition [77]. Amu, Fajobi [78] analyzed the combined effect of lime and ESP on improving the bearing capacity of the soil. The maximum improvement was seen by utilizing 7% lime with ESP under unsoaked and soaked conditions. Similar improvements in the CBR value by utilizing ESP were also reported by other studies [22,79,80]. The study conducted by Naseem, Mumtaz [66] utilized tire rubber powder (TRP) up to 5%. The CBR value improved from 1.02% to 5.1% by utilizing 5% TRP. Similar phenomena have been observed in other studies, showing that adding TRP to soil increases its bearing capacity [81,82]. When combined with expansive soil, these materials act synergistically to enhance particle binding, reduce swell potential, and increase load-bearing capacity.

## 4. Statistical Evaluation of ESP and RTP Effects

The statistical results for the ESP and RTP treatment series are presented in Table 4. The parameters that did not satisfy the normality assumption and the corresponding alternative statistical tests are summarized in Table 5. Levene’s test was nonsignificant for all evaluated parameters, indicating no evidence of unequal variance among the treatment groups. Residual normality was satisfied for most parameters. However, departures from normality were identified for PI and 14-day UCS in the ESP series and for OMC, unsoaked CBR, and soaked CBR in the RTP series. These parameters were therefore evaluated using the Kruskal–Wallis test, whereas the remaining parameters were analyzed using one-way ANOVA. Post-hoc comparisons were conducted only when the corresponding overall statistical test was significant.

The primary objective of the statistical analysis was to evaluate whether ESP and RTP dosages resulted in significant differences in measured soil properties among treatment groups. The statistical results show that ESP dosage had statistically significant effects on most mechanical parameters. The mean PI decreased with increasing ESP content, indicating an improvement in soil plasticity; however, this effect was not statistically significant at the replicate level (Kruskal–Wallis, *p* = 0.3538, ε^2^ = 0.045). Similarly, both LL and PL showed statistically significant improvements, with large effect sizes of η^2^ = 0.862 and 0.952, respectively, indicating that ESP strongly influences the plasticity characteristics of expansive soil. Both MDD and OMC showed statistically significant effects, with large effect sizes of η^2^ = 0.939 and 0.921, respectively, confirming that ESP influenced the compaction behavior of the soil. This suggests that while ESP affects density, its influence on moisture requirements during compaction is limited. The UCS improvement was statistically significant after 28 days (*p* = 0.0002, η^2^ = 0.834), whereas the effects at 0 and 14 days were not statistically significant, indicating that the cementitious nature of calcium carbonate in ESP contributes more clearly to long-term strength gain. Swelling potential was also significantly reduced across all curing durations, with large effect sizes ranging from η^2^ = 0.983 to 0.992, demonstrating ESP’s effectiveness in minimizing soil expansion. Furthermore, both unsoaked and soaked CBR values improved significantly, with large effect sizes of η^2^ = 0.938 and 0.970, respectively, confirming that ESP contributes directly to load-bearing capacity. Overall, these results establish ESP as a highly effective stabilizer, especially in improving strength, reducing plasticity, and controlling swelling behaviour.

Similarly, the results in Table 4 reveal significant effects of RTP on several soil mechanical properties. The statistical analysis shows that RTP had a notable influence on the consistency limits, with PL exhibiting a large effect size (η^2^ = 0.894), suggesting that RTP incorporation improves soil workability by reducing excessive plasticity. The LL and PI also showed statistically significant responses, with effect sizes of η^2^ = 0.851 and 0.661, respectively, confirming that RTP influenced the plasticity characteristics of the treated soil. In terms of compaction, both MDD and OMC showed statistically significant treatment effects. MDD exhibited a large effect size (η^2^ = 0.908), while OMC was significant under the Kruskal–Wallis test (*p* = 0.0300, ε^2^ = 0.744), confirming that RTP influenced both dry density and moisture requirements during compaction. For strength development, the effect of RTP on UCS was statistically significant after 28 days (*p* = 0.0332, η^2^ = 0.645), whereas the effects at 0 and 14 days were not statistically significant. The significant 28-day response reflects RTP’s role in improving longer-term strength through enhanced particle interlocking. The most striking effect of RTP was observed in the reduction in swelling potential, with large effect sizes ranging from η^2^ = 0.985 to 0.989 across all curing periods. This indicates that RTP is particularly effective in mitigating the expansive behavior of soil. Finally, both unsoaked and soaked CBR values were significantly influenced, with effect sizes of ε^2^ = 0.808 and 0.833, respectively, confirming the improvement in load-bearing performance. Overall, RTP proved highly effective in reducing swelling potential and improving load-bearing and compaction performance, while a statistically significant effect on UCS became evident after 28 days.

## 5. Comparative Evaluation of Engineering Properties

Table 6 presents a comparative summary of the geotechnical properties and percentage changes in soil treated with ESP and RTP. As shown in the table, the incorporation of ESP and RTP reduced the Atterberg limits, the optimum moisture content, and the soil’s swelling potential, indicating improved volumetric stability and reduced plasticity. In contrast, MDD, UCS, and CBR show a significant increase when ESP and RTP are added to the unreinforced soil sample. However, approximately 20% ESP and 6% RTP were generally found to provide optimal performance. Beyond this range, particularly around 9% RTP content, the rubber particles’ beneficial effects on flexibility and ductility were offset by reduced maximum dry density and weaker interparticle bonds, resulting in decreased performance. However, excessive or unbalanced additive proportions may reduce stabilization efficiency and lead to inconsistent engineering behavior.

Although swelling potential decreased slightly more at 9% RTP than at 6% RTP at 28 days, with reductions of 85.7% and 80.9%, respectively, the higher RTP content reduced the improvement in all other measured properties. The PI reduction decreased from 24.2% at 6% RTP to 18.2% at 9% RTP, while the MDD improvement decreased from 27.1% to 18.5%. Strength results also showed a similar trend, with the 28-day UCS improvement decreasing from 32.6% to 22.5%, and the CBR results following the same trend. The unsoaked CBR improvement decreased from 174.0-fold to 165.2-fold, and the soaked CBR improvement decreased from 300.0-fold to 283.3-fold, both relative to the untreated soil. These results show that adding RTP beyond 6% yields only a marginal further reduction in swelling, but at the cost of reduced compaction, strength, plasticity, and bearing performance. This behavior is likely due to excess rubber content, which can reduce particle packing efficiency and weaken interparticle bonds within the stabilized soil matrix. Therefore, 20% ESP + 6% RTP was selected as the optimum blend because it provided the best overall response across the evaluated parameters.

## 6. Stabilization Mechanism of ESP and RTP Treated Soil

**Figure 11:**
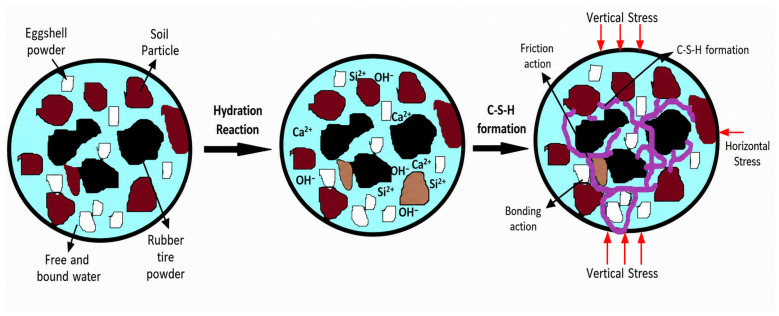
Interaction mechanism of soil with ESP and RTP.

Figure 11 illustrates the three-stage interaction mechanism among soil, ESP, and RTP. In the first stage, ESP, RTP, and soil particles coexist in the presence of free and bound water. When water is introduced, calcium carbonate in ESP dissolves, releasing calcium ions (Ca^2+^) and hydroxyl ions (OH^−^) into the pore water and raising the soil pH. Under these alkaline conditions, calcium ions replace sodium and potassium ions on clay mineral surfaces via cation exchange, reducing the thickness of the diffuse double layer around clay particles and causing them to clump into larger aggregates [20,83]. At the same time, the higher pH breaks down reactive silica (Si^2+^) and alumina from clay surfaces, as shown in the middle stage of Figure 11. These released ions then react with calcium to form calcium silicate hydrate (C-S-H) and calcium aluminate hydrate (C-A-H), which act as binding agents that hold soil particles together [84].

RTP plays a distinct yet equally important role in this process. Unlike ESP, RTP does not participate in chemical reactions. Instead, it acts as a physical reinforcement within the soil [60]. As shown in the right stage of Figure 11, rubber particles from RTP fill voids created within the ESP-modified soil fabric and form an interlocking network throughout the matrix. This network contributes in two ways: it creates friction between rubber particles and soil grains, and it allows the C-S-H network to bond around both soil and rubber particles [85]. The result is a soil that resists both vertical and horizontal stresses more effectively, as shown in Figure 11, and is far less prone to cracking and deformation under load. The porous surface of RTP particles also retains moisture within the soil for longer, which keeps the alkaline environment active and allows ESP-driven cementitious reactions to continue [66].

The true benefit of combining ESP and RTP becomes clear when the results are compared with those for each additive used alone. ESP alone raised the 28-day UCS from 178 kPa to 217 kPa and reduced swelling potential by 61.9%. When 6% RTP was added to the optimum ESP dosage, UCS increased to 238 kPa and swelling potential dropped by 80.9%. This additional gain cannot be explained by RTP alone, since RTP has no chemical reactivity [60]. What actually happens is that ESP first alters the soil chemistry and restructures the clay fabric into a more open, aggregated arrangement. RTP then locks into that restructured fabric and reinforces it mechanically through friction and interlocking [85]. Put simply, ESP builds the chemical foundation and RTP strengthens the physical structure on top of it. This is why the combined treatment consistently outperforms either material used on its own across all measured parameters [19,83,84].

## 7. Environmental, Sustainability, and Economic Implications

From environmental, sustainability, and economic perspectives, the use of ESP and RTP in soil stabilization offers important advantages by promoting the beneficial reuse of waste materials that would otherwise require disposal and long-term management. Their utilization supports waste minimization and resource recovery while reducing reliance on conventional stabilizing agents, which are often associated with greater environmental burdens and higher material costs. To quantitatively assess economic viability, material costs were compared under local market conditions in Pakistan. Conventional stabilizers such as cement and lime are currently priced at approximately USD 115/ton and USD 90–100/ton, respectively. By contrast, the raw material cost of ESP and RTP is negligible, with processing expenses for grinding, sieving, and drying estimated at USD 5–15/ton. At the optimum treatment dosage of 20% ESP and 6% RTP, approximately 343 kg of additives are required per cubic meter of treated soil, resulting in an estimated stabilization cost of USD 0.7–1.9/m^3^. This compares favorably with conventional cement stabilization at a typical 5% dosage, which costs approximately USD 7.6/m^3^, representing a potential cost reduction of 75–90%. Therefore, the comparison demonstrated that substituting waste-derived ESP and RTP for conventional chemical stabilizers yielded substantial cost savings without compromising engineering performance. Accordingly, the proposed approach is both economically feasible and practically feasible for large-scale ground improvement projects, particularly in areas where disposal of these waste materials is an increasingly pressing issue.

## 8. Limitations and Future Work

This study evaluated the short-term engineering behavior of ESP- and RTP-stabilized expansive soil over a maximum curing period of 28 days under controlled laboratory conditions. While the results demonstrate clear and statistically significant improvements in strength, plasticity, and volumetric stability, several durability-related aspects were not addressed and represent important limitations of the present work. No wet–dry cycling or freeze–thaw testing was conducted, both of which are critical for assessing the long-term performance of stabilized soils under seasonal field conditions. Repeated wetting and drying cycles can progressively degrade the cementitious bonds formed by ESP, while freeze–thaw cycles may loosen the interlocking structure provided by RTP particles. Without such testing, the durability of the stabilized soil under field exposure conditions remains uncertain. Additionally, RTP may undergo aging-related degradation, and the leaching behavior of ESP and zinc-containing RTP under prolonged environmental exposure was not assessed. Future studies should include wet–dry and freeze–thaw durability testing, long-term leaching assessments, and comprehensive heavy metal characterization to evaluate the environmental safety of RTP-treated soils. Microstructural characterization using SEM, XRD, and FTIR is also recommended to further validate the proposed stabilization mechanisms. Field-scale trials would ultimately be necessary to confirm that the laboratory performance observed in this study translates into reliable long-term behavior under real-site conditions.

## 9. Conclusions

This study demonstrated that eggshell powder (ESP) and recycled tire powder (RTP) can significantly enhance the strength, stability, and overall engineering performance of expansive soil.

(1) The optimal combination of 20% ESP and 6% RTP led to a substantial reduction in soil plasticity, with the plasticity index (PI) decreasing from 33% to 25%, which improves soil workability and reduces susceptibility to deformation.
(2) Compaction characteristics were also enhanced, as the maximum dry density (MDD) increased from 1.32 g/cm^3^ to 1.81 g/cm^3^ and the optimum moisture content (OMC) decreased from 23% to 20% for the ESP + RTP-treated soil, while ESP-only treatment decreased the OMC to 18%, facilitating improved densification behavior. Furthermore, ANOVA showed that ESP significantly affected OMC (*p* < 0.0001), confirming that ESP addition influenced the moisture requirements of the soil during compaction.
(3) Unconfined Compressive Strength (UCS) showed a notable improvement, rising from 162 kPa to 238 kPa over 28 days, confirming increased load-bearing capacity.
(4) Swelling potential was significantly reduced at both 6% and 9% RTP (80.9% and 85.7% after 28 days). The slightly greater swelling reduction at 9% RTP came with lower gains in MDD (27.1% vs. 18.5%), 28-day UCS (32.6% vs. 22.5%), and CBR (unsoaked increases of 174.0% vs. 165.2%; soaked increases of 300.0% vs. 283.3%). The optimal mixture was therefore 20% ESP + 6% RTP, based on the best overall balance among compaction, strength, plasticity, swelling, and CBR, not the maximum swelling reduction alone.
(5) California Bearing Ratio (CBR) values also improved markedly, with unsoaked CBR reaching 6.3% and soaked CBR increasing to 4.8%, indicating enhanced performance for pavement applications.
(6) Statistical analysis using one-way ANOVA and the Kruskal–Wallis test confirmed significant effects of ESP and RTP on most measured soil properties, supporting the reliability of the experimental findings.

Overall, the results confirm that ESP and RTP can effectively improve the geotechnical and pavement-related performance of expansive soils under short-term laboratory conditions. Their combined use provides a practical stabilization approach for enhancing strength, compaction behavior, and volumetric stability in problematic soil deposits, though long-term durability aspects such as wet–dry cycling and freeze–thaw resistance should be addressed in future studies before field-scale implementation.

## Figures and Tables

**Table 1:** Physical parameters of the expansive soil.

Parameters	Expansive Soil	Standard
% Gravel	0	ASTM Code D6913 [37]
% Sand	1.9	ASTM Code D6913 [37]
% Fines	98.1	ASTM Code D7928 [38]
Soil Classification (AASHTO)	A-7-6	ASTM Code D3282 [39]
Soil Classification (USCS)	CH	ASTM Code D2487 [40]
Liquid Limit, LL (%)	62	ASTM Code D4318 [41]
Plastic Limit, PL (%)	29	ASTM Code D4318 [41]
Plastic Index, PI (%)	33	ASTM Code D4318 [41]
Unconfined compressive strength, UCS (kPa)	162	ASTM Code D2166 [42]
Maximum dry density, MDD (g/cm^3^)	1.32	ASTM Code D698 [43]
Optimum moisture content, OMC (%)	23%	ASTM Code D698 [43]
Swelling Potential, SP (%)	63	ASTM Code D4546 [44]
California bearing ratio for unsoaked (%)	2.3	ASTM Code D1883 [45]
California bearing ratio for soaked (%)	1.2	ASTM Code D1883 [45]

**Table 2:** Chemical parameters of the expansive soil and ESP.

Parameters	Soil (%)	ESP (%)
SiO_2_	56.9	2.2
Al_2_O_3_	22.4	1.7
FeO	11.2	-
CaO	3.9	78.4
P_2_O_5_	-	1.3
TiO_2_	2.2	-
MgO	1.4	2.6
Na_2_O	0.4	11.1
Others	1.6	2.7

**Table 3:** Physical and chemical composition of rubber tire powder.

Parameters	Description	Standards
**Physical**		
Appearance	Black color fines	-
Specific gravity	1.08	ASTM D792 [47]
Solubility	Insoluble	ASTM D 185 [48]
Softening Point (°C)	165–170	ASTM D36 [49]
Density (g/cm^3^)	0.82	ASTM D1895 [50]
**Chemical**		
Rubber	56%	ASTM D297 [51]
Carbon black	27%	
Sulfur	2%	
Zinc	1%	
Other additives	14%	

**Table 4:** Statistical analysis of soil mechanical parameters for the ESP and RTP treatment series.

Parameters	Statistical Test	df	Test Statistic	Effective Size (η^2^)	*p*-Value	Significant Effect
ESP						
LL	OW ANOVA	F = 15.008	15.008	η^2^ = 0.862	0.0001	Yes
PL	OW ANOVA	F = 47.429	47.429	η^2^ = 0.952	<0.0001	Yes
PI	Kruskal–Wallis	H = 5.538	5.538	ε^2^ = 0.045	0.3538	No
MDD	OW ANOVA	F = 36.870	36.87	η^2^ = 0.939	<0.0001	Yes
OMC	OW ANOVA	F = 28.099	28.099	η^2^ = 0.921	<0.0001	Yes
UCS (0 days)	OW ANOVA	F = 1.423	1.423	η^2^ = 0.372	0.2846	No
UCS (14 days)	Kruskal–Wallis	H = 10.637	10.637	ε^2^ = 0.470	0.0591	No
UCS (28 days)	OW ANOVA	F = 12.029	12.029	η^2^ = 0.834	0.0002	Yes
Swelling potential (7 days)	OW ANOVA	F = 140.938	140.938	η^2^ = 0.983	<0.0001	Yes
Swelling potential (14 days)	OW ANOVA	F = 239.900	239.9	η^2^ = 0.990	<0.0001	Yes
Swelling potential (21 days)	OW ANOVA	F = 295.430	295.43	η^2^ = 0.992	<0.0001	Yes
Swelling potential (28 days)	OW ANOVA	F = 263.889	263.889	η^2^ = 0.991	<0.0001	Yes
Unsoaked CBR	OW ANOVA	F = 36.540	36.54	η^2^ = 0.938	<0.0001	Yes
Soaked CBR (28 days)	OW ANOVA	F = 76.474	76.474	η^2^ = 0.970	<0.0001	Yes
RTP						
LL	OW ANOVA	3, 8	F = 15.232	η^2^ = 0.851	0.0011	Yes
PL	OW ANOVA	3, 8	F = 22.551	η^2^ = 0.894	0.0003	Yes
PI	OW ANOVA	3, 8	F = 5.190	η^2^ = 0.661	0.0279	Yes
MDD	OW ANOVA	3, 8	F = 26.240	η^2^ = 0.908	0.0002	Yes
OMC	Kruskal–Wallis	3	H = 8.949	ε^2^ = 0.744	0.0300	Yes
UCS (0 days)	OW ANOVA	3, 8	F = 0.687	η^2^ = 0.205	0.5851	No
UCS (14 days)	OW ANOVA	3, 8	F = 3.677	η^2^ = 0.580	0.0625	No
UCS (28 days)	OW ANOVA	3, 8	F = 4.838	η^2^ = 0.645	0.0332	Yes
Swelling potential (7 days)	OW ANOVA	3, 8	F = 231.408	η^2^ = 0.989	<0.0001	Yes
Swelling potential (14 days)	OW ANOVA	3, 8	F = 177.931	η^2^ = 0.985	<0.0001	Yes
Swelling potential (21 days)	OW ANOVA	3, 8	F = 176.082	η^2^ = 0.985	<0.0001	Yes
Swelling potential (28 days)	OW ANOVA	3, 8	F = 190.503	η^2^ = 0.986	<0.0001	Yes
Unsoaked CBR	Kruskal–Wallis	3	H = 9.462	ε^2^ = 0.808	0.0237	Yes
Soaked CBR (28 days)	Kruskal–Wallis	3	H = 9.667	ε^2^ = 0.833	0.0216	Yes

Note: For one-way ANOVA, F denotes the test statistic, df is reported as between-group and within-group degrees of freedom, and η^2^ represents the effect size. For the Kruskal–Wallis test, H denotes the test statistic, df represents the number of groups minus one, and ε^2^ represents the effect size. Statistical significance was evaluated at *p* < 0.05.

**Table 5:** Assessment of statistical assumptions and selection of appropriate tests for the ESP and RTP treatment.

Soil Mechanical Parameters	Additive	Shapiro–Wilk *p*-Value	Levene *p*-Value	Assumptions Satisfied	Statistical Test Used
PI	ESP	0.0466	0.9522	No	Kruskal–Wallis
UCS (14 days)	ESP	0.0330	0.9957	No	Kruskal–Wallis
OMC	RTP	0.0367	0.9978	No	Kruskal–Wallis
Unsoaked CBR	RTP	0.0395	0.9961	No	Kruskal–Wallis
Soaked CBR	RTP	0.0197	0.9711	No	Kruskal–Wallis

Note: Parameters listed here failed normality and were analyzed using the Kruskal–Wallis test. All others satisfied the assumptions and were analyzed using one-way ANOVA.

**Table 6:** Summary of Geotechnical Properties and Percentage Changes in Treated Soil.

Parameter		Soil + 5% ESP	Soil + 10% ESP	Soil + 15% ESP	Soil + 20% ESP	Soil + 25% ESP	Soil + 20% ESP + 3% RTP	Soil + 20% ESP + 6% RTP	Soil + 20% ESP + 9% RTP
LL	%	−4.8	−9.6	−12.9	−17.7	−16.1	−22.6	−29.0	−27.4
		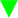	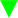	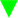	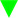	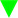	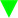	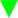	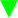
PL	%	−6.8	−17.2	−20.6	−27.5	−20.6	−24.1	−34.4	−37.9
		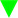	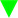	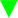	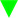	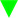	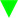	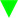	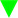
PI	%	−3.0	−3.0	−6.0	−9.0	−12.1	−21.2	−24.2	−18.2
		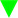	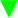	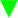	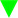	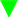	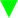	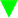	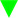
MDD	%	6.8	10.6	13.2	16.9	14.3	21.4	27.1	18.5
		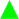	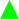	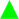	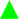	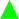	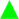	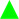	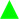
OMC	%	4.0	−4.0	−4.0	−13.0	−21.6	4.0	−13.0	8.6
		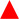	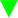	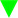	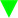	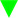	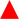	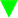	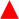
UCS-0 day	%	2.5	3.1	4.3	7.4	−0.6	8.6	12.3	10.5
		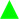	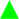	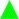	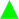	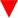	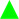	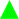	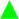
UCS-14 day	%	5.9	8.9	10.7	14.2	5.9	19.5	26.6	17.2
		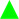	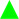	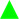	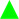	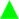	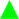	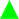	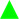
UCS-28 day	%	3.4	10.7	14.1	21.9	16.7	24.2	32.6	22.5
		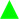	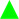	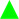	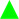	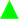	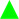	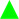	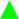
Percent swell-7 day	%	−11.1	−36.5	−41.3	−50.8	−65.1	−69.8	−74.6	−82.5
		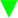	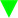	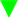	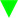	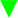	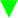	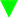	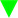
Percent swell-14 day	%	−14.3	−41.3	−44.4	−55.5	−68.3	−71.4	−76.2	−82.5
		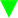	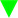	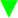	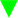	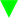	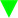	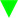	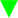
Percent swell-21 day	%	−19.1	−46.0	−50.1	−60.3	−69.8	−74.6	−77.8	−84.1
		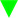	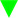	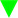	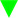	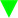	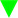	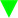	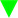
Percent swell-28 day	%	−22.2	−47.6	−53.9	−61.9	−71.4	−76.2	−80.9	−85.7
		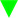	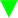	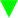	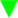	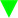	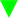	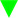	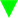
CBR (unsoaked)	times	13.0	34.7	65.2	78.3	69.6	121.0	174.0	165.2
		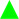	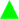	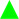	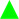	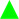	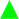	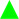	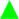
CBR (soaked)-28 days	times	41.6	116.6	158.3	191.6	166.6	241.6	300.0	283.3
		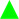	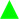	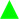	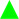	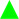	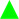	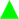	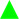

Note: The percentage changes reported for RTP-treated samples were calculated relative to the optimum ESP-treated soil specimen (20% ESP). 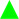 Increase (positive impact), 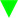 Decrease (positive impact), 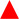 Increase (negative impact), 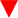 Decrease (negative impact).

## Data Availability

Data are contained within the article.
